# Library instruction in medical education: a survey of current practices in the United States and Canada

**DOI:** 10.5195/jmla.2018.374

**Published:** 2018-01-02

**Authors:** Amanda M. Nevius, A’Llyn Ettien, Alissa P. Link, Laura Y. Sobel

## Abstract

**Objective:**

The most recent survey on instruction practices in libraries affiliated with accredited medical institutions in the United States was conducted in 1996. The present study sought to update these data, while expanding to include Canadian libraries. Additional analysis was undertaken to test for statistically significant differences between library instruction in the United States and Canada and between libraries affiliated with highly ranked and unranked institutions.

**Methods:**

A twenty-eight-question survey was distributed to libraries affiliated with accredited US and Canadian medical schools to assess what and how often librarians teach, as well as how librarians are involved in the curriculum committee and if they are satisfied with their contact with students and faculty. Quantitative data were analyzed with SAS, R, and MedCalc.

**Results:**

Most of the seventy-three responding libraries provided instruction, both asynchronously and synchronously. Library instruction was most likely to be offered in two years of medical school, with year one seeing the most activity. Database use was the most frequently taught topic, and libraries reported a median of five librarians providing instruction, with larger staffs offering slightly more education sessions per year. Libraries associated with highly ranked schools were slightly more likely to offer sessions that were integrated into the medical school curriculum in year four and to offer sessions in more years overall.

**Conclusions:**

In US and Canadian libraries, regardless of the rank of the affiliated medical school, librarians’ provision of instruction in multiple formats on multiple topics is increasingly common.

## INTRODUCTION

Studies dating back to the 1930s have shown libraries playing a role in medical education [1], and a well-maintained library with staff responsive to a school’s needs is a requirement for accreditation of medical schools by the Liaison Committee on Medical Education (LCME) [2]. Historically, libraries have addressed this requirement of responsiveness by providing instruction on efficient access to medical information, with the rationale that students who are able to quickly locate and retrieve relevant information will have more time to evaluate and absorb this information and will better retain it during and after their courses and exams.

To investigate how and how often such library instruction is delivered to medical students, Earl conducted a survey of 123 US medical school libraries in 1996 [3], and Eldredge et al. conducted a 2013 regional update, surveying 17 medical school libraries in the western United States [4]. These 2 surveys showed that instructional content that libraries provided covered topics such as use of literature databases and citation management software, professional writing, and critical evaluation and was provided in a variety of formats including workshops, lectures, virtual instruction, and orientation sessions. While providing valuable information, the 1996 study is now outdated, and the 2013 update did not cover the entire United States. Therefore, the authors conducted a new survey with 2 initial aims: (1) to update the original Earl study and (2) to expand its coverage to include Canada, as both US and Canadian medical schools are accredited by the LCME (Canadian schools are accredited jointly by the LCME and the Committee on the Accreditation of Canadian Medical Schools).

While analyzing the survey data, we also became interested in identifying any differences in library instruction between libraries affiliated with medical schools in the United States and Canada or between libraries affiliated with highly ranked and unranked medical schools. While we expected there to be no differences between US and Canadian libraries, we hypothesized that more highly ranked medical schools would be more likely to have libraries reporting a high level of participation in the medical school, with the logic that highly ranked medical schools would be more likely to value and incorporate interprofessional education.

## METHODS

The 2014 LCME list of accredited medical schools in the United States and Canada [5] was consulted to identify a survey population of 157 accredited schools (there were 158 accredited schools in 2014, but 1 was accidentally left off the list by the research team). A search of each school’s library website was performed, and best efforts were made to identify a contact who was responsible for education programming. During this process, 2 pairs of schools were found to share libraries (4 schools and 2 libraries in total), leaving 155 contacts. One library listed no electronic contact information and was not sent a survey. Therefore, the total number of contacts was 154.

Consulting previous research, we developed a twenty-eight-question survey with a mixture of qualitative and quantitative questions (supplemental Appendix A). To ensure compliance with institutional guidelines, a draft was sent to Boston University’s Institutional Review Board, which ruled the project exempt on the basis that it was not human subjects research. Google Forms was selected as the survey platform. The survey began with an initial statement of risks and benefits as well as a definition of six terms used in the questions (asynchronous library instruction, synchronous library instruction, formal library instruction sessions, informal library instruction sessions, curriculum-integrated library instruction, non-curriculum-integrated library instruction).

The survey was sent to contacts via email on December 17, 2014. Reminder emails were sent on January 13, 2015, and January 29, 2015, with the official close date listed as January 31, 2015. The survey was left open for late responses until February 4, 2015. After the close date, data were reviewed to identify if responses needed clarification, and if respondents had provided contact information, we emailed them to request more details. Unclear portions of responses were discarded if no contact information was given or if the contacted librarian did not reply.

Geographic demographic groups were identified using US Census Bureau and Canadian Parliament regional divisions [6, 7]. As no school-ranking instruments rated both the United States and Canada without also ranking worldwide institutions, the QS World University Rankings 2014 were chosen as the school ranking instrument, based on its reputation for avoiding English-language bias and focusing on quality of instruction [8]. Because this instrument ranks only the top 200 schools, respondents were split into those that were ranked in the top 200 (T200) and those that were unranked.

Qualitative responses were analyzed by researchers, who assigned general categories such as “clarification” or “added information” and evaluated them for trends and insight into quantitative responses. Quantitative data were analyzed with SAS, R, and MedCalc statistical software using the “N-1” chi-squared test of proportions, Spearman’s rank-order correlation, and Fisher’s exact test.

## RESULTS

### Response rate and demographics

We received a total of 74 responses from the 154 libraries associated with LCME-accredited medical schools in the United States, US territories, and Canada to which the survey was sent. One response, which lacked identifying information for a specific library, was discarded. While not all responses to all questions were quantifiable, every remaining response contained usable data, leaving 73 records for consideration and resulting in a response rate of 47%.

Responding libraries represented 33 US states (out of 45 with LCME-accredited schools), Puerto Rico, and 6 Canadian provinces (out of 8 with LCME-accredited schools). There was no significant difference in the response rate of Canadian and US libraries, although the majority of responses (86%, n=63 out of 73 unless otherwise stated) came from the United States and US territories due to the greater number of surveyed libraries in this area. Responses from each region roughly paralleled the total number of surveyed libraries in each region (supplemental Appendix B provides detailed regional response rates). There was also no significant difference in response rate between libraries affiliated with T200 institutions (30%, n=22 out of 73) and unranked institutions, although the majority of responses came from unranked institutions due to their greater representation in the survey population.

### Delivery and initiation of library instruction

We found that most responding libraries provided instruction that was intended to be used in an asynchronous manner (i.e., accessed by users at their convenience), with 95% of libraries (n=69) providing instruction through using email or web forms, 85% (n=62) creating subject guides or LibGuides, and 78% (n=57) creating recorded tutorials. Use of synchronous instruction (i.e., in-person, remote classes, or real-time instruction over the phone) was also very common, with 97% of libraries (n=71) offering face-to-face instruction sessions, 70% (n=51) providing instruction over the phone, and 52% (n=38) using instant messaging (IM) or chat software. While remote meeting software and videochat were relatively uncommon, at 32% (n=23) for remote meeting and 25% (n=18) for videochat software, the 2 were frequently found together, with 72% (n=13 out of 18) of those using videochat software also reporting using remote meeting software. We intended these categories to differentiate between synchronous computer- or mobile device–based communication programs with and without a video component, but it is possible that the categories might not have been distinct for respondents.

Synchronous instruction was usually initiated in response to curriculum requirements (i.e., library sessions presented as part of a formal course), with 82% (n=60) of libraries reporting sessions taking place in this context, though faculty- and student-initiated informal instruction (i.e., teaching sessions offered on a drop-in basis or instructional reference desk interactions) were also commonly reported, at 75% (n=55) and 73% (n=53), respectively. A notable minority (8%, n=6) also reported some version of “librarian-initiated” instruction in the free-text “other” category, suggesting that a number of librarians take it upon themselves to organize and offer instructional sessions.

Most respondents (86%, n=63) provided orientation for new students, with attendance usually mandatory (81%, n=51 out of 63). A smaller majority (62%, n=45) offered orientation for new faculty members, with attendance usually optional (82%, n=37 out of 45).

Most respondents (90%, n=66) offered instructional sessions integrated into the medical school curriculum (i.e., part of a formal course offered by the school) in at least 1 academic year, most commonly year 1: 64% (n=47) reported integrated sessions, not including orientation, in this year. Instruction was integrated into years 2 and 3 by equal numbers of libraries, with 48% (n=35) reporting integration in each of these years, while year 4 was the least likely to include any library instruction in the curriculum, with only 11% (n=8) reporting any curriculum-integrated instructional activity.

Almost one-third of respondents (32%, n=23) offered curriculum-integrated instruction in only 1 year, most often year 1 (57%, n=13 out of 23), followed by year 3 (26%, n=6 out of 23) and year 2 (17%, n=4 out of 23). Slightly more respondents (37%, n=27) reported integration in 2 different years, most commonly year 1 and year 3 (33%, n=9 out of 27) or year 1 and year 2 (30%, n=8 out of 27). A smaller number of respondents (16%, n=12) offered integrated material in 3 different years, and only 4% (n=3) reported curriculum-integrated material in all 4 years of the standard undergraduate medical education.

### Curriculum committee

A majority of respondents (75%, n=55) indicated that at least 1 librarian had some level of involvement with the medical school’s curriculum committee. Qualitative responses elaborating on this often indicated that the participating librarian was the library director and clarified the level of participation, ranging from newly acquired ex-officio status to long-standing voting membership.

### Frequency and type of instruction sessions per academic year

In addition to instruction in each year of medical school, the survey asked for the number of times per academic year that librarians taught classes in a variety of categories. Medians are reported due to significant outliers in all categories. Curriculum-integrated library instruction was offered a median of 4 times (range, 0–78) per academic year, whereas non-curriculum-integrated sessions (i.e., instruction not linked to a formal course, such as an optional research skills session) were offered a median of 3 times (range, 0–90) per academic year.

**Figure 1:**
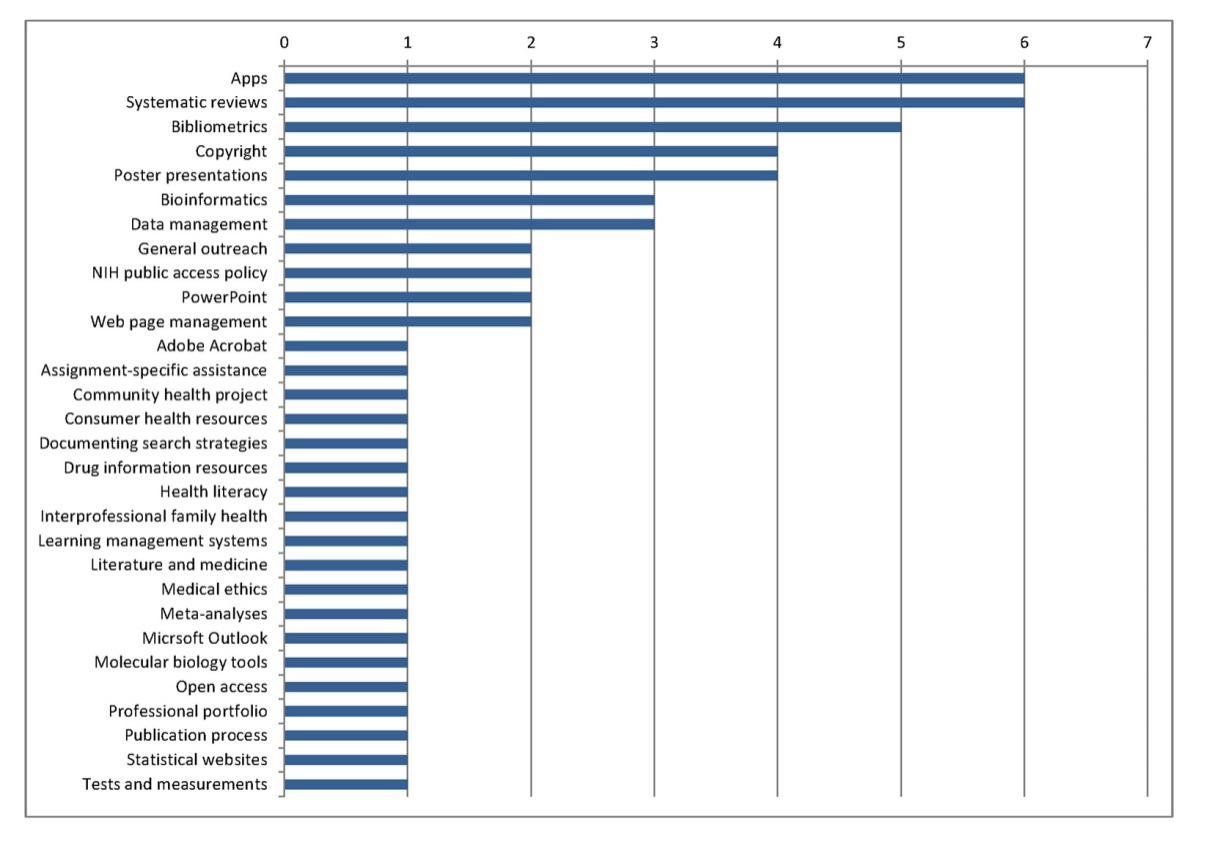
Other category: instruction sessions

Databases were the most frequently taught subject, with a median of 5 (range, 0–128) per academic year. Evidence-based medicine (EBM) was taught in a median of 4 (range, 0–60) sessions, and citation management and information literacy a median of 1 each (range, 0–40 and 0–147, respectively). Respondents were also given the option to write in other subjects taught at their libraries. Although the majority (62%, n=45) reported none, giving this question a median of 0 (range, 0–69), 28 did indicate other types of instruction. Of these, 27 specifically listed the different courses taught, showing a wide range of topics with the most frequently reported being a tie between apps and systematic reviews (Figure 1).

### Staff size

Respondents reported a median of 5 librarians participating in library instruction (range, 1–17). A weak-to-moderate positive correlation between larger staffs and greater frequency of teaching was detected using Spearman’s rank-order correlation (n=73; Table 1). This relationship was true for all instruction categories, with the exception of sessions on information literacy. We found no significant correlations between the size of the education staff and responses to any other questions.

**Table 1:** Education staff size and frequency of teaching in various categories

	Curriculum-integrated	Non-curriculum-integrated	Databases	Evidence-based medicine (EBM)	Citation management software	Information literacy	Other topics
Correlation coefficient	0.318	0.417	0.440	0.358	0.434	0.195	0.234
*p*-value	0.006	0.0002	<0.0001	0.001	0.0001	0.097	0.046

### Desired levels of curriculum integration and faculty and student contact

Most respondents (85%, n=62) reported that they would prefer more integration into the medical school curriculum, and while 15% (n=11) were satisfied with their current level of integration, none desired less integration. Similarly, most respondents expressed a preference for more contact with medical school faculty (70%, n=51) and students (73%, n=53), and no respondent desired less contact with either group. This was reflected in the qualitative responses, with many comments stating the desire for more contact and more integration: “[R]eally, doesn’t everyone yearn for ‘more integration’ no matter what?”

However, even the positive responses mentioned concerns, with those indicating satisfaction with their level of involvement often mentioning time as a limiting factor or noting that greater integration and more contact would require a larger staff: “We have almost reached the point of not being able to take on additional instruction due to time and staffing.” The third year of the medical curriculum was the most frequently mentioned as one in which respondents would like greater contact and/or integration: “We have great integration into the preclinical years but our third year is in flux right now.”

### Top 200 versus unranked institutions

Using the “N-1” chi-squared test of proportions for the 2 categories (schools ranking in the top 200 versus those not ranked), we found that 41% of libraries affiliated with T200 schools were integrated into 3 or more years of the medical school curriculum, compared with 14% of libraries affiliated with unranked institutions, a difference of 26% (χ^2^=6.993, *df*=1, *p*=0.0082). Looking at specific library services, we found that 100% of T200 schools offered asynchronous subject guides or LibGuides, compared with 78% of unranked institutions, a difference of 22% (χ^2^=5.641, *df*=1, *p*=0.0175). No significant relationships were detected between school ranking and responses to any other questions.

### US versus Canadian institutions

Using Fisher’s exact test, we found that Canadian libraries were more likely to report integration into the curriculum in year 1 (*p*=0.037) and more likely to report being satisfied with their existing level of integration (*p*=0.011). No significant relationships were detected between geographical location and responses to any other questions.

### Comparison to Earl and Eldredge

While asking different questions overall, both Earl’s survey and our survey investigated whether library instruction was a required part of the curriculum and whether formal library instruction was offered. Using “N-1” chi-squared test of proportions for these 2 categories (Earl’s survey respondents versus the current survey respondents), we found that 36% of Earl’s respondents indicated that library instruction was required in the medical curriculum, whereas 82% of current survey respondents reported the same, a difference of 46% (χ^2^=28.019, *df*=1, *p*<0.0001). Similarly, 75% of Earl’s respondents reported offering formal library instruction, while 95% of the current survey’s respondents offered these sessions, a difference of 20% (χ^2^=10.599, *df*=1, *p*=0.0011).

Where questions overlapped, our results tended to agree with those of Eldredge et al., suggesting that practices in libraries in the western United States are similar to those in other regions where medical schools are accredited by the LCME. As we did, Eldredge et al. found curriculum committee involvement to be common and student orientation to be very common and usually mandatory (12 of 13 respondents to Eldredge et al. reported required student orientation). They found that curriculum-integrated library sessions were most common in year 1 of the medical curriculum, followed by year 3 and year 2, with little reported activity in year 4, which paralleled our results.

Unlike Eldredge et al., we did not inquire separately about the number of virtual and face-to-face sessions, so direct comparison was impossible, but their finding that face-to-face sessions (i.e., hands-on sessions and lectures) were almost 5 times as common as virtual sessions (“which included work through blogs, online student peer assessment, wikis, videos or online tutorials”) also roughly paralleled our results. We found that 78% of libraries offered recorded tutorials and 52% used IM or chat software, while of Eldredge et. al.’s 8 respondents reporting what they called “virtual” instruction represented 8 individual libraries from their 13 respondents, this would be 62%. It is possible that our results show a slight growth in this area since 2010, but given the imperfect overlap in the questions asked, this cannot be stated with confidence. Further studies examining face-to-face and virtual instruction in detail could shed more light on this question.

## DISCUSSION

Our survey responses indicate that library instruction is integrated into the curriculum most frequently in year 1 and least frequently in year 4. While supported by Eldredge et al.’s study, this contrasts with Miller’s 2004 survey of libraries that were affiliated with US accredited medical schools, which found that library courses offered for credit are most common in year 1 (45.5%) and year 4 (27.3%) [9]. While the surveys asked different questions of different populations, the variance regarding year 4 is still notable. It is possible that noncredit library courses are more common in years 2 and 3 and were not counted by Miller, while the majority of year 4 courses were offered for credit. Since our survey did not differentiate between for-credit and noncredit library instruction, more targeted research would be needed to resolve this question.

Libraries conduct instruction both asynchronously and synchronously, showing attention to different learning styles and needs [10]. However, some research shows a student preference for “learning via online-asynchronous modes” [11], suggesting that increased focus on asynchronous options may be appropriate. While our results could indicate some increase in such sessions in the short time between Eldredge et al.’s study and our study, suggesting that education librarians are aware of this growing demand, the data cannot be confidently interpreted on this point.

Both student and faculty orientations are offered by a majority of libraries, though faculty orientations are somewhat less common and are usually optional rather than mandatory. Studies of student orientation are well represented in the literature, with reports on redesigning these sessions, using them to gather data on students, or needing to expand beyond orientation [11–15]. Our results support the presence of student orientations as a critical “entry point” for student instruction.

Faculty orientations are not discussed in the literature, which is perhaps not surprising given our findings on the less common and often optional nature of such sessions. Considering the strong tendency among respondents to desire increased contact with faculty, orientation sessions may warrant consideration as an overlooked venue for outreach to new faculty members. On the other hand, it is possible that libraries that are not currently offering such sessions have discontinued them after limited success in the past. Minimal research has been done in this area, but Gardner et al.’s study of library web pages directed toward faculty suggests that trainings of any kind promoted specifically to faculty are uncommon [16]. Because there may not be the same administrative support for requiring faculty attendance that there is for students, these sessions, even if offered, may be poorly attended at many institutions—a question this survey did not investigate. Further research could shed more light on this question and clarify whether faculty orientations could be a valuable access point for librarians who are attempting to increase their faculty contact.

Respondents teach about databases most frequently, reflecting a professional understanding, summarized by Chen, that “it is a responsibility of librarians to guide students in understanding how to search databases efficiently” [17]. Teaching the use of databases is also highlighted by Lynn as part of the Association of American Medical Colleges Medical School Objectives Project [18]. However, the literature also shows that librarians desire to move beyond pure database instruction, and this is reflected in our responses—both in the annual frequency of EBM and citation management sessions and in the number of write-in “other” responses when participants were asked for the topics of instruction sessions.

There is some disagreement in the literature as to whether these types of instruction fall into the traditional domain of librarians and are, therefore, librarians’ professional responsibility [19], or whether, as “related nonlibrary services” [20], they represent useful information that is not strictly necessary for library users in the purest sense of enabling them to make use of libraries and are, therefore, above and beyond librarians’ professional duty. In either case, perceived needs are being addressed, and a measurable service is being provided to medical schools and their students and faculty. Notably, while a relatively small number of institutions wrote in “apps” as an instructional topic, a survey of 4 Canadian institutions by Boruff and Storie [21] indicate that 42.7% of users desire training in this area, and Stokes, Light, and Haines’s [22] report on an instructional program for library-supported apps shows that education on this subject can be well received by students. Some institutions are obviously recognizing this emergent need, but perhaps more librarians could consider whether its value to their users might justify attention, even given the challenges involved in keeping up with a rapidly changing field.

A majority of respondents reported a librarian on the curriculum committee, with qualitative analysis indicating that this is usually perceived as valuable. These findings accord with the well-documented value of committee participation in the literature, where it is usually reported to be an important means of promoting the integration of library skills and information literacy into the curriculum [23–29]. Some of our respondents did highlight other means of supporting library integration into the curriculum, suggesting that while committee membership is valuable, it is neither absolutely necessary nor the only way to approach participation in curriculum development:

While we don’t have a librarian on the committee, we have a librarian working with different departments to schedule librarian instruction during specific topics or research modules, thus making the instruction tie in with curriculum

On curriculum committee *and* part of med ed departmental meetings. (These are much more valuable—usually by the time something gets to curriculum committee it’s pretty well developed).

Others noted that mere “involvement” with the curriculum committee may not be meaningful if library input is not sought:

The director attends the meetings but plays no part in the…roles of the librarians. Communication from the curriculum committee back to the librarians is insufficient

Having a librarian on the Curriculum Committee is…a vast improvement over the previous exclusion. However, current involvement is passive and there is no possibility to become involved with administrators/faculty debating and making the actual decisions.

Our results indicate that most education librarians desire more contact with both faculty and students, with a minority being satisfied with their status quo and none desiring less contact. This reflects a common understanding that it is more difficult to meaningfully instruct and assist students with limited contact [10]. Interestingly, survey respondents indicated a slight preference (3%) for contact with students over faculty. While this difference is too small to be meaningful in itself, the fact that there is not a greater desire for increased contact with faculty contradicts literature indicating that greater faculty contact is the best way to increase meaningful interactions with both students and faculty [30, 31].

Current medical education strategies highlight integrated learning and interprofessional education, with vertical integration working to prevent disconnected silos of information by referencing different disciplines in multiple locations throughout the curriculum (particularly referencing clinical work in preclinical years and basic sciences in clinical years). The integration of library instruction into multiple years, including both preclinical and clinical years, suggests it is being treated with the same attention shown to other subject areas in the medical curriculum. Similarly, any meaningful work with librarians can be considered interprofessional and can help demonstrate to students the value of referring to other professions when providing quality care [32].

While we hypothesized that libraries that are affiliated with highly ranked medical schools would report greater integration and/or more frequent instructional sessions, this is only weakly supported by the results, which show limited correlation between school rankings and integration into the curriculum or services provided, and no relationship between ranking and frequency of teaching. Because libraries that are affiliated with highly ranked medical schools are more likely to report integration into three or more years of the medical curriculum, it is possible that while the total number of teaching sessions has no necessary relationship to excellence in medical education, the timing of that instruction throughout the curriculum may.

However, our findings suggest that medical schools regardless of rank dedicate a baseline level of support to their libraries, as required by the LCME, and that there is no strong trend toward greater use of librarians or library instructional services among highly ranked schools. Considering medical education at all ranks, comparisons to Earl’s study indicate that formal integrated library instruction has increased by 20% and library instruction as a requirement in the curriculum has increased by 46% since 1996. This greater presence in formal education indicates that library instruction is increasingly viewed as a key part of the medical education experience than an optional add-on, by institutions at all levels.

There has historically been limited comparison of academic library instruction in the United States and Canada [33, 34], but what exists has tended to show minimal differences along “largely parallel paths” [35]. This survey’s findings are in keeping with these results, suggesting that librarians in academic medical libraries engage in comparable instructional practices in the United States and Canada. Our findings that Canadian librarians are both more likely to be satisfied with their level of integration into the medical school curriculum and more likely to be integrated in year one may highlight the importance of the first year (perhaps integration in year one is sufficiently valuable that it is associated with increased satisfaction). However, we do not observe this correlation when examining all responses regarding year one and ideal integration, and because no other responses show significant regional differences, it seems likely that these results (though reported for completeness) are essentially random.

Two prior surveys examining staff size and library instruction have shown no significant correlations. Miller, surveying all libraries affiliated with accredited US medical institutions regarding librarian-offered credit courses, has found no association between staff size and the number of credit courses offered [9], and Joubert and Lee, analyzing thirty-five Association of Academic Health Sciences Libraries (AAHSL) participating in the LibQUAL+ survey, have found no correlation between staff size and overall quality of service [36]. These surveys indicate that quality and for-credit instruction are not impacted by staff size.

The current survey did not differentiate between for-credit and noncredit courses and made no attempt to assess quality, but we were somewhat surprised that the overall frequency of instruction appeared to be only modestly impacted by staff size. It may seem intuitively obvious that more education librarians would be able to teach more often, but our respondents included libraries with small education staffs offering many instructional sessions, along with larger staffs providing fewer instructional sessions, so it was likely that institutional focus and individual job description were as important as numbers in determining how many instructional sessions that librarians offer per year. We also asked generally for the number of librarians who participated in library instruction, and several respondents noted that their counts included those working part-time on education, so it was possible that if we had asked specifically for full-time equivalent (FTE) numbers, a stronger correlation might have appeared.

This study has several limitations. Our survey was not tested for clarity prior to being sent out, and although definitions of terms were provided, it was possible that survey takers interpreted them differently, skewing results toward more or less frequent provision of various types of topics for instruction. The preselection by survey writers of certain types of library instruction might have forced an artificial categorization of instruction on respondents. Finally, an overall number of instruction sessions per academic year was not requested, nor was the number of library instruction sessions for credit, and we did not define “education staff” in a way that allowed definitive analysis of staffing and teaching patterns.

Results of this survey of libraries affiliated with accredited medical schools in the United States and Canada show an ever greater integration of library instruction into the medical school curriculum, occurring at comparable levels in both countries. Librarians’ adaptability and attention to user needs is seen in the frequency of instruction, both in more traditional library instruction areas such as databases and in newer ones such as apps. Education librarians show adaptation to modern educational formats, offering instruction both asynchronously and synchronously, though student preferences might justify even more asynchronous instruction sessions. While a majority of respondents desire greater integration and contact with students and faculty, the fact that both of these result from contact with faculty is perhaps not thoroughly understood, as the desire for greater faculty contact is no higher than the desire for greater student contact.

While larger staffs appear to teach slightly more sessions per academic year, our analysis indicates that a large education staff is not a requirement for frequent instruction, although the survey did not investigate other job responsibilities that librarians held or the amount of time that individual librarians devote to the education program. The 20% increase in institutions offering formal library instruction over the past 2 decades highlights the importance of the instructional library staff in fulfilling a recognized need in the medical school curriculum, indicating that education librarians provide a valued service in medical education.

## Supplemental Files

Appendix A Text of survey instrument

Appendix B Geographic breakdown of responses
