# Supplementary material for: Library instruction in medical education: a survey of current practices in the United States and Canada
Source: J Med Libr Assoc. 2018 Jan 2;106(1):98–107. doi: 10.5195/jmla.2018.374 (PMC5764599; doi:10.5195/jmla.2018.374)
Supplement: Appendix A [file jmla-106-98-s001.pdf]

JMLA Journal of the Medical Library Association

SC

Supplemental content to
J Med Libr Assoc. Jan;106(1):dx.doi.org/10.5195/jmla.2018.374
www.jmla.mlanet.org
© Nevius, Ettien, Lin, Sobel 2018

Library instruction in medical education: a survey of current practices in the United States and Canada

Amanda M. Nevius; A'Llyn Ettien, AHIP; Alissa P. Link; Laura Y. Sobel

APPENDIX A

Text of survey instrument

Integration of library instruction into medical education in the United States and Canada

Thank you for participating in our survey!

This eighteen-question survey is being sent to libraries affiliated with all of the accredited medical schools in the United States and Canada in order to ascertain to what extent libraries are a part of medical school education in these countries.

The survey should be completed by the person most familiar with the education program in your library. If you are not that person, we would appreciate your forwarding it to the correct recipient.

The survey will be active until January 23, 2015. Please complete it at your earliest convenience. We will send reminder emails before the close date to those institutions from which we have not received responses.

Participation in this survey is voluntary and imposes no responsibilities on you. You may discontinue the survey at any time, and no information will be collected until you click "submit" on the last page.

We estimate it should take no more than 10 minutes to complete the survey.

RISKS of participation:
We foresee no significant risks to you as a result of completion of this survey. Results will be anonymous unless you choose to give your name, and no information gathered will ever be reported with a link to your name. It is possible that we may mention results for your specific institution, and that even though your name will not be directly linked with these results, someone could guess that you were the person who responded to the survey. While we do not anticipate this posing a problem, if you perceive any personal or professional risk from such a connection, you should not complete the survey.

BENEFITS of participation:
We foresee no immediate benefits to you as a result of completing this survey. We hope to formally report results following compilation and review of the information gathered, and the information you share may be used to help better understand the types of education services offered to medical schools by libraries in the United States and Canada.

This survey has been approved by the Institutional Review Board of Boston University.

If you have any questions, please feel free to contact Amanda McNeil, education and information services librarian, at [email address removed].

Definitions of terms we use:

- Asynchronous library instruction: Instructional materials produced by the library and intended to be accessed by users at their convenience (i.e., recorded tutorials, written FAQs, subject guides/LibGuides, etc.)
- Synchronous library instruction: Real-time instruction either in person or remotely (i.e., via Skype, chat, remote meeting software, etc.)

SC
Supplemental content to
J Med Libr Assoc. Jan;106(1):dx.doi.org/10.5195/jmla.2018.374
www.jmla.mlanet.org
© Nevius, Ettien, Lin, Sobel 2018

- Formal library instruction sessions: Library instruction sessions requested by faculty or administration, or offered by the library at a scheduled time
- Informal library instruction sessions: Library instruction sessions offered on a drop-in basis, appointments, and reference desk interactions
- Curriculum-integrated library instruction: Library instruction offered as part of a course listed in the formal medical school curriculum, within course meeting time (i.e., one class period dedicated to library orientation in an evidence-based medicine [EBM]–focused course)
- Non-curriculum-integrated library instruction: Library instruction offered outside the formal medical school curriculum (i.e., an optional research skills session scheduled outside of a course's normal meeting hours)

1. Please identify your library and the medical school(s) affiliated with it.

2. How many librarians at your library participate in asynchronous or synchronous library instruction for the medical school(s)?

3. Please check off all asynchronous library instruction your institution offers to the medical school(s).
- Email/Web forms/Ask a librarian
- Tutorials
- Subject guides/LibGuides
- Other

4. Please check off all synchronous library instruction your institution offers to the medical school(s).
- Face-to-face
- Video chatting software (i.e., Skype)
- IM/Chat
- Phone
- Remote meeting/Reference software
- Other

5. For synchronous library instruction, please indicate how the instruction is initiated. (Check all that apply.)
- Medical school curriculum–initiated formal library instruction
- Faculty-initiated formal library instruction
- Student-initiated informal library instruction
- Faculty-initiated informal library instruction

6. Is a librarian involved with your institution's curriculum committee?
- Yes
- No

7a. Is this level of librarian involvement in the curriculum committee satisfactory for your library?
- Yes
- No

7b. Please elaborate on your previous answer if desired.

8a. Does the library offer new faculty orientation sessions for the medical school?
- Yes
- No

8b. If yes, is attendance mandatory for new medical faculty members? (Leave blank if answer to previous question is "No.")
- Yes
- No

9a. Does the library offer new student orientation sessions for the medical school?
- Yes
- No

9b. If yes, is attendance mandatory for new medical students? (Leave blank if answer to previous question is "No.")
- Yes
- No

10. If library instruction is integrated into the medical school curriculum, during which years is it required? (Check all that apply.)
- Year 1 (not including orientation)
- Year 2
- Year 3
- Year 4

Definitions of terms we use:
- Curriculum-integrated library instruction: Library instruction offered as part of a course listed in the formal medical school curriculum, within course meeting time (i.e., one class period dedicated to library orientation in an EBM-focused course)
- Non-curriculum-integrated library instruction: Library instruction offered outside the formal medical school curriculum (i.e., an optional research skills session scheduled outside of a course's normal meeting hours)
- Formal library instruction sessions: Library instruction sessions requested by faculty or administration, or offered by the library at a scheduled time
- Informal library instruction sessions: Library instruction sessions offered on a drop-in basis, appointments, and reference desk interactions

11. Approximately how often do library staff teach curriculum-integrated formal library instruction sessions for medical students per academic year? (Please give your best estimate: give your answer as a specific number, i.e., "5" or "five," NOT a term like "often" or "rarely.")

12. Approximately how often do library staff teach non-curriculum-integrated formal library instruction sessions for medical students per academic year? (Please give your best estimate: give your answer as a specific number, i.e., "5" or "five," NOT a term like "often" or "rarely.")

Supplemental content to
J Med Libr Assoc. Jan;106(1):dx.doi.org/10.5195/jmla.2018.374
www.jmla.mlanet.org
© Nevius, Ettien, Lin, Sobel 2018

13a. Databases: Please indicate how often formal library instruction sessions covering databases are offered per academic year. (Please give your best estimate: give your answer as a specific number, i.e., "5" or "five," NOT a term like "often" or "rarely.")
13b. EBM: Please indicate how often formal library instruction sessions covering evidence-based medicine are offered per academic year. (Please give your best estimate: give your answer as a specific number, i.e., "5" or "five," NOT a term like "often" or "rarely.")
13c. Citation management: Please indicate how often formal library instruction sessions covering the use of citation management software are offered per academic year. (Please give your best estimate: give your answer as a specific number, i.e., "5" or "five," NOT a term like "often" or "rarely.")
13d. Information literacy: Please indicate how often formal library instruction sessions covering general information literacy are offered per academic year. (Please give your best estimate: give your answer as a specific number, i.e., "5" or "five," NOT a term like "often" or "rarely.")
13e. Other: If library staff teach subjects not covered by this list, please include them here, and indicate how often they are taught per academic year. (Please give your best estimate: give your answer as a specific number, i.e., "5" or "five," NOT a term like "often" or "rarely.")

14a. Compared to what your library currently has, what would be the ideal level of integration into the medical school curriculum?

- Less integration
- The same amount of integration
- More integration

14b. Please elaborate on your previous answer if desired.

15a. Compared to what your library currently has, what would be the ideal level of medical school faculty contact?

- Less contact
- The same amount of contact
- More contact

15b. Please elaborate on your previous answer if desired.

16a. Compared to what your library currently has, what would be the ideal level of medical student contact?

- Less contact
- The same amount of contact
- More contact

16b. Please elaborate on your previous answer if desired.

17. Is there anything else you would like to add?

18. If we have any questions about your responses, would it be all right to contact you? If "yes," please provide your preferred contact information. (If "no," leave blank.)
