# Supplementary material for: Library instruction in medical education: a survey of current practices in the United States and Canada
Source: J Med Libr Assoc. 2018 Jan 2;106(1):98–107. doi: 10.5195/jmla.2018.374 (PMC5764599; doi:10.5195/jmla.2018.374)
Supplement: Appendix B [file jmla-106-98-s002.pdf]

Journal of the Medical Library Association

Supplemental content to
J Med Libr Assoc. Jan;106(1):dx.doi.org/10.5195/jmla.2018.374
www.jmla.mlanet.org
© Nevius, Ettien, Lin, Sobel 2018

Library instruction in medical education: a survey of current practices in the United States and Canada

Amanda M. Nevius; A'Llyn Ettien, AHIP; Alissa P. Link; Laura Y. Sobel

APPENDIX B

Geographic breakdown of responses

US Census Bureau regional division	Number of responses from region	Number of medical school libraries in region (US total 139)	Percentage of US responses	Percentage of total US medical school libraries	Response rate of region
Midwest	18	34	29%	24%	53%
Northeast	13	33	21%	24%	39%
South	25	51	40%	37%	49%
West	5	17	8%	12%	29%
Puerto Rico*	2	4	3%	3%	50%

* Puerto Rico, a US territory, is counted in the US total although not part of a US Census region.

Canadian Parliament division	Number of responses from region	Number of medical school libraries in region (Canadian total 17)	Percentage of Canadian responses	Percentage of total Canadian medical school libraries	Response rate of region
Maritimes	1	1	10%	6%	100%
Newfoundland and Labrador	1	1	10%	6%	100%
Ontario	4	6	40%	35%	67%
Quebec	2	4	20%	24%	50%
Western	2	5	20%	29%	40%
